# Synergetic Sensing Effect of Sodium Carboxymethyl Cellulose and Bismuth on Cadmium Detection by Differential Pulse Anodic Stripping Voltammetry

**DOI:** 10.3390/s19245482

**Published:** 2019-12-12

**Authors:** Jingheng Ning, Xin Luo, Faxiang Wang, Shouen Huang, Jianhui Wang, Dongmin Liu, Donglin Liu, Donger Chen, Jiaqian Wei, Yongle Liu

**Affiliations:** School of Chemistry and Food Engineering, Changsha University of Science & Technology, Changsha 410110, China; luoxin_gl@126.com (X.L.); wfaxiang@163.com (F.W.); huangshouen@csust.edu.cn (S.H.); wangjh0909@csust.edu.cn (J.W.); dongminkl@126.com (D.L.); dong993@163.com (D.L.); chendonger9@163.com (D.C.); Weijiaqian02@163.com (J.W.)

**Keywords:** sodium carboxymethyl cellulose, bismuth, differential pulse anodic stripping voltammetry, cadmium detection

## Abstract

In the present work, a novel electrochemical sensor was developed for the detection of trace cadmium with high sensitivity and selectivity in an easy and eco-friendly way. Firstly, a glassy carbon electrode (GCE) was modified with nontoxic sodium carboxymethyl cellulose (CMC) by a simple drop-casting method, which was applied to detect cadmium by differential pulse anodic stripping voltammetry (DPASV) in a solution containing both target cadmium and eco-friendly bismuth ions, based on a quick electro-codeposition of these two metal ions on the surface of the modified electrode (CMC-GCE). Investigated by scanning electron microscopy (SEM), energy dispersive spectroscopy (EDS), and Fourier transform infrared spectroscopy (FT-IR), both CMC (with good film-forming ability) and bismuth (with well-defined stripping signal) were found to be well complexed with target cadmium, leading to vital signal amplification for cadmium detection at a sub-nanomolar level. Under the optimal conditions, the proposed sensor exhibited a good linear stripping signal response to cadmium (Ⅱ) ion, in a concentration range of 0.001 μmol/L–1 μmol/L with a limit of detection of 0.75 nmol/L (S/N = 3). Meanwhile, the results demonstrate that this novel electrochemical sensor has excellent sensitivity and reproducibility, which can be used as a promising detection technique for testing natural samples such as tap water.

## 1. Introduction

Nowadays, wide applications of heavy metal such as cadmium, chrome, lead, arsenic, and mercury, are causing serious problems including environmental pollution and risks to human health [1]. Among them, cadmium ions might be the most common and toxic, and can be found almost everywhere in water/soil or even in air, mainly due to its extensive uses in electroplating industries [2,3]. Once cadmium ions enter the food chain, they will be absorbed by the living body and accumulate in many organs such as lungs, liver, kidneys, and bones, hard to remove yet easily damaging these organs [4,5]. The terrible itai-itai disease event that occurred in Japan is a typical example of cadmium poisoning, caused by cadmium contaminated rice [6]. Other kinds of diseases owing to cadmium poisoning, such as renal insufficiency and cancer, have also been reported from very early research and have aroused growing public concerns [7]. In view of cadmium’s high toxicity, the World Health Organization has stipulated clearly in its publication, Guideline for Drinking Water Quality, that the maximum concentration for cadmium contaminants in drinking water should not exceed 0.0266 μmol/L [8]. For many years, researchers have been working on the development of all kinds of techniques for the removal and detection of cadmium pollutants [9,10,11,12]. To supervise cadmium residues in our surroundings at any time or any place, it is very urgent to develop new and highly efficient methods for the determination of trace cadmium in easy and quick ways.

Generally, conventional analytical methods for the detection of heavy metal ions include atomic absorption spectrometry (AAS) [13], atomic fluorescence spectrometry (AFS) [14], inductively coupled plasma mass spectrometry (ICP-MS) [15], or inductively coupled plasma atomic emission spectrometry (ICP-AES) [16]. However, these methods have obvious limitations such as sophisticated instrumentation, long operation times, and high running cost [17]. Comparatively, the electrochemical analysis method seems to be more attractive and practicable due to its remarkable sensitivity, simplicity, and low cost [18]. Among the electrochemical methods, differential pulse anodic stripping voltammetry (DPASV) has been successfully used to detect heavy metal ions, which is usually based on special chemically-modified electrodes [19,20]. For a long time, mercury-modified electrodes, mainly including hanging drop mercury and thin mercury film electrodes, were the typical chemically modified electrodes, as they showed good results in stripping analysis of heavy metals [21,22]. However, mercury is toxic and polluting and actually is not suitable for wide application, especially in view of the tide of green chemistry. Thus, researchers have been working hard to look for eco-friendly substitutes with excellent analytical performances to replace mercury-based electrodes. Consequently, due to low toxicity as well as other merits such as easy preparation, well-defined stripping signal, and wide negative potential window, bismuth-based electrodes that can form a fused alloy with a heavy metal [23,24], have attracted much attention since first reported in 2000 as a desirable mercury-electrode substitute for the detection of heavy metal ions [25,26,27,28]. Moreover, it has been widely reported that the introduction of metal oxides/nanoparticles or carbon nanomaterials into a bismuth-based sensing platform can enlarge stripping signals, since their synergetic effects can promote the accumulation of target metal ions upon the electrode surface [29,30,31].

Sodium carboxymethyl cellulose (CMC), the most widely used and the largest type of cellulose in the world, has many merits such as easy availability and low cost, high hydrophilicity and biocompatibility, nontoxicity and environmental friendliness, and so on [32]. As a common polysaccharide derived from natural cellulose, CMC possesses a large number of hydroxyl and carboxymethyl groups in its polymer chains, which can effectively chelate and so adsorb many kinds of metal ions [33,34,35]. Besides, its innate viscosity endows CMC with an outstanding film-forming ability, which makes it suitable for the modification of the electrode surface as a functional film material [36]. In the construction of lithium batteries, CMC has been reported to act as a binder and help active particles to deposit uniformly on the surface of the electrode, due to its great influence on the accumulation of particles onto electrode surface [37]. More recently, CMC has been reported to successfully improve the peak-current response when combined with graphene oxide and glutathione so as to realize the simultaneous detection of cadmium and lead ions [38]. Therefore, CMC-based electrode might be another promising choice to replace the environment-hazardous mercury-modified electrode for the detection of heavy metal ions.

**Scheme 1:**
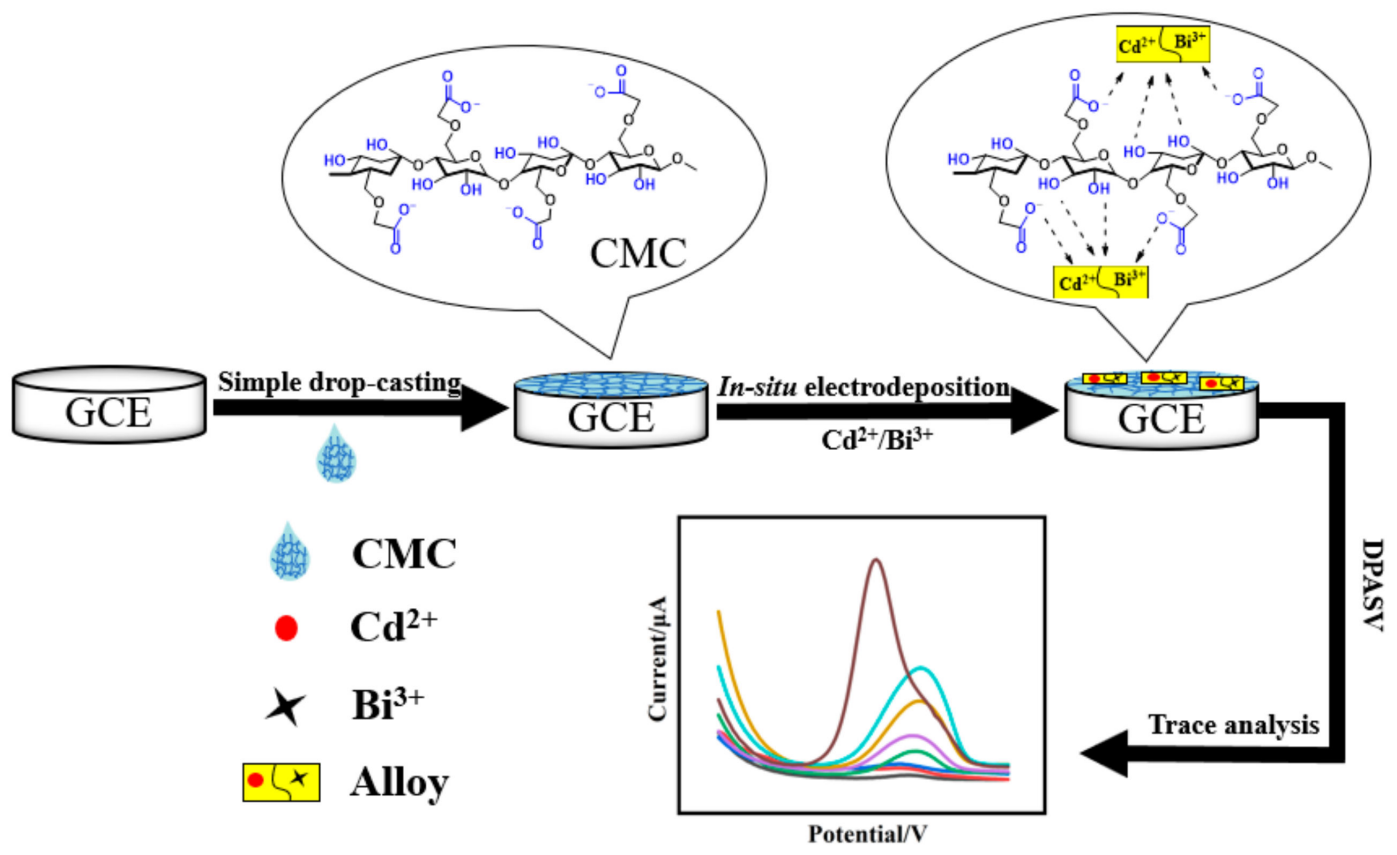
Strategy for the construction of the new cadmium electrochemical sensor based on synergetic effects of both CMC and bismuth on the enrichment and detection of cadmium ions.

Herein, in this study, we fabricated a new electrochemical sensor through modifying the most common glassy carbon electrode (GCE) with CMC, followed by in situ deposition of bismuth (Ⅲ) (Bi^3+^) on the surface of CMC-modified GCE for the first time, aiming at highly sensitive determination of trace cadmium (Ⅱ) (Cd^2+^) by DPASV. The strategy for the construction of this new cadmium sensor is shown in Scheme 1. Firstly, CMC can be easily assembled onto the GCE surface only by a simple drop-casting method due to its good film-forming ability, to form a CMC-modified electrode (CMC-GCE). Secondly, both bismuth ions and the target cadmium ions in the sample solution can be simultaneously in situ electrodeposited onto the surface of the CMC-GCE, to construct a new sensor based on bismuth and CMC modified GCE (Bi/CMC-GCE). Attributed to the synergetic effect between bismuth (able to form fused alloy with cadmium ion) and CMC (capable of chelating cadmium ions with lots of hydroxyl and carboxyl groups), the anodic stripping signal can be greatly amplified to the extent that it is enough for sensitive detection of trace Cd^2+^. At the same time, this cadmium sensor has other outstanding merits of the two modification materials, such as a well-defined stripping signal and wide negative potential window of bismuth as well as easy obtainment and nontoxicity of CMC. Therefore, the successful fabrication of this new sensor based on Bi/CMC-GCE will develop an easy and quick, simple and sensitive, low-cost and eco-friendly electrochemical detection method for the analysis of trace cadmium residue in our surroundings. This work may open and pave potential avenues towards for various applications in the field of detection of heavy metal ions.

## 2. Experimental

### 2.1. Chemicals and Reagents

All chemicals and reagents were of analytical grade and all of the water used in this study was produced by the ultra-pure water equipment (Eco-S system, 18.2 MΩ cm^−1^, Hi-tech Instruments Co., Ltd., Shanghai, China). Sodium carboxymethyl cellulose, acetic acid, sodium acetate trihydrate, cadmium nitrate, bismuth nitrate pentahydrate, lead nitrate, copper nitrate, zinc nitrate, iron(III) chloride, cobaltous acetate, aluminum nitrate nonahydrate, manganese acetate, iron(II) chloride, and nickel nitrate hexahydrate were purchased from Sinopharm Chemical Reagent Co., Ltd. (Shanghai, China), and were used without further purification. 

### 2.2. Apparatus

The electrochemical measurements were performed on a CHI660E electrochemical workstation instrument (Shanghai Chenhua Instrument Co., Ltd., Shanghai, China), using a conventional three-electrode system with a modified glassy carbon electrode (3 mm diameter) as the working electrode, a platinum wire as the counter electrode, and an Ag/AgCl electrode as the reference electrode. 

The scanning electron microscope (SEM) and energy dispersive spectroscopy (EDS) were performed on a Hitachi S-4800 microscope (Hitachi, Tokyo, Japan). The Fourier transform infrared spectra (FT-IR) of CMC, the complexes CMC-Cd and CMC-Cd-Bi were recorded from 4000 cm^−1^ to 400 cm^−1^ by a Thermo Nicolet iS10 infrared spectrometer (Nicolet Instrument Co., Ltd., Madison, WI, USA). 

### 2.3. Preparation of the Working Electrode

Firstly, the bare GCE was polished with 0.3 μm and 0.05 μm alumina slurries, and then was successively treated with ultra-pure water, ethanol, and ultra-pure water for 5 min by ultrasonic washing (50 W, 40 kHz), and afterwards was dried in air. CMC was dispersed in ultra-pure water and was ultra-sonicated for 15 min to give a CMC solution. The modified electrode was prepared by a simple drop casting method, carefully dripping 5 μL of CMC solution onto the surface of the pre-treated GCE, and then dried at room temperature.

### 2.4. Electrochemical Measurements 

Electrochemical measurements of Cd^2+^ were performed at room temperature by DPASV, with Cd^2+^ and Bi^3+^ in NaAc/HAc buffer solution (0.1 mol/L, pH 4.5). Cd^2+^ was reduced at a certain deposition potential for enough time under stirring, to make metal ions deposited on the electrode surface as much as possible. Then, after equilibrated for 2 s without stirring, DPASV curves were recorded in the potential range from −1.0 to −0.6 V with a frequency of 25 Hz, amplitude of 25 mV, and step potential of 5 mV. Finally, the work electrode was cleaned at 0.3 V for 60 s under stirring to remove cadmium residual and bismuth film. Each scanning was repeated three times and all experiments under the optimized experimental conditions. CMC solution: 0.4 mg/mL; bismuth ion solution: 0.5 μmol/L; pH 4.5; deposition potential: −1.2 V; accumulation time: 180 s; scan rate: 50 mV·s^−1^.

### 2.5. Detection of Cadmium Ion in Natural Samples 

In order to demonstrate the applicability and reliability of the method for natural samples, firstly a series of cadmium solutions with a concentration range of 0.01–0.1 μmol/L were prepared just using tap water to dissolve cadmium nitrate, and then were detected by DPASV under the optimal conditions.

## 3. Results and Discussion

### 3.1. Investigation on the CMC, CMC-Cd and CMC-Cd-Bi by FT-IR

**Figure 1:**
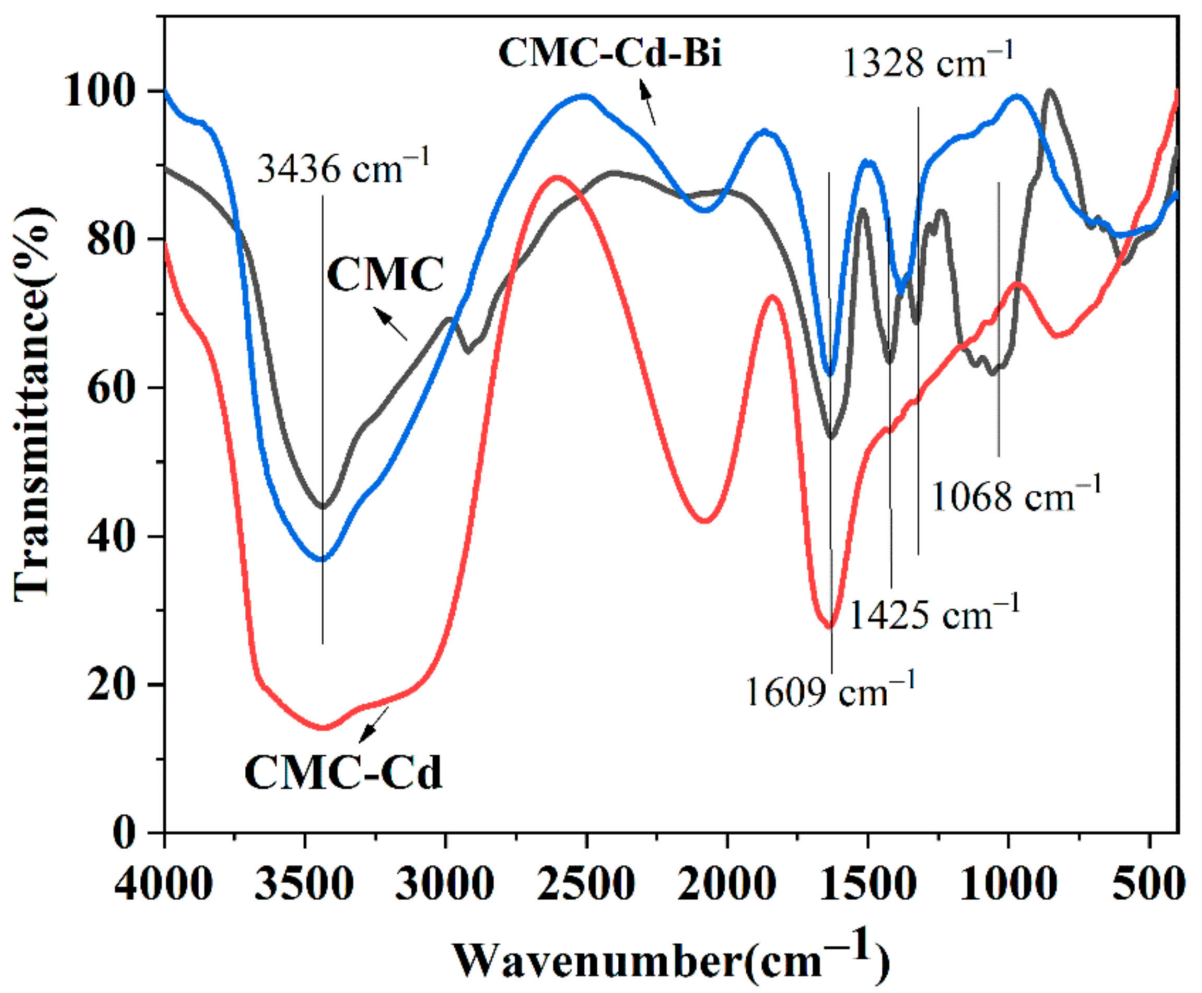
FT-IR spectra of sodium carboxymethyl cellulose (CMC) and its complexes CMC-Cd and CMC-Cd-Bi.

To study the combination ability of CMC with cadmium ions, the infrared spectra of CMC and its composite with Cd (II) (CMC-Cd) were obtained, as shown in Figure 1. The spectrum of CMC displays specified peaks at 3436, 1609, 1425, 1328, 1068, and 892 cm^−1^. The broad and strong band from 3436 cm^−1^ to 2500 cm^−1^ can be attributed to O–H stretching vibration for strong well-known hydrogen bonds caused by lots of hydroxyl and carboxyl groups in CMC. The band at 1609 cm^−1^ can be attributed to the C=O asymmetric vibrations in carboxyl groups. The bands at 1425 and 1328 cm^−1^ are related to symmetric stretching vibration of alkyl groups in CMC. The peaks at both 1068 and 892 cm^−1^ represent C–O–C stretching vibrations. As for the spectra of CMC-Cd and CMC-Cd-Bi, the peak intensity of the bands at 1068 and 1328 cm^−1^ can hardly be observed, compared to that of CMC. These results demonstrate that CMC has good ability to interact with cadmium ions by coordination bond, suggesting the possible adsorption and enrichment of cadmium onto the surface of the CMC-modified electrode.

### 3.2. Investigation on the Synergetic Effects of Both CMC and Bismuth on the Enrichment of Cadmium by SEM and EDS

**Figure 2:**
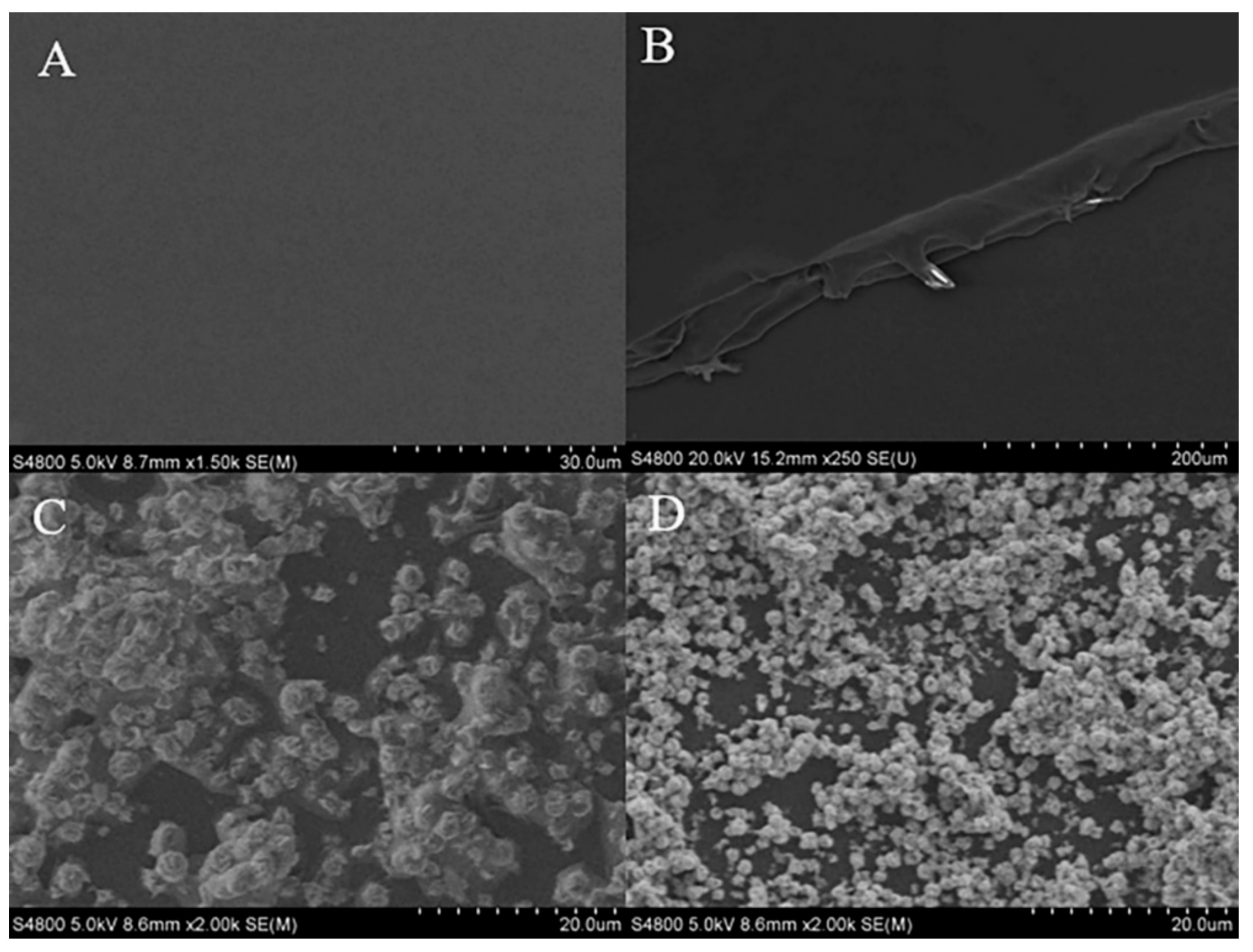
SEM images of the bare glassy carbon electrode (GCE) (**A**), CMC-GCE (**B**), bare GCE after electrodeposition of cadmium and bismuth (**C**), and CMC-GCE after electrodeposition of cadmium and bismuth (**D**).

**Figure 3:**
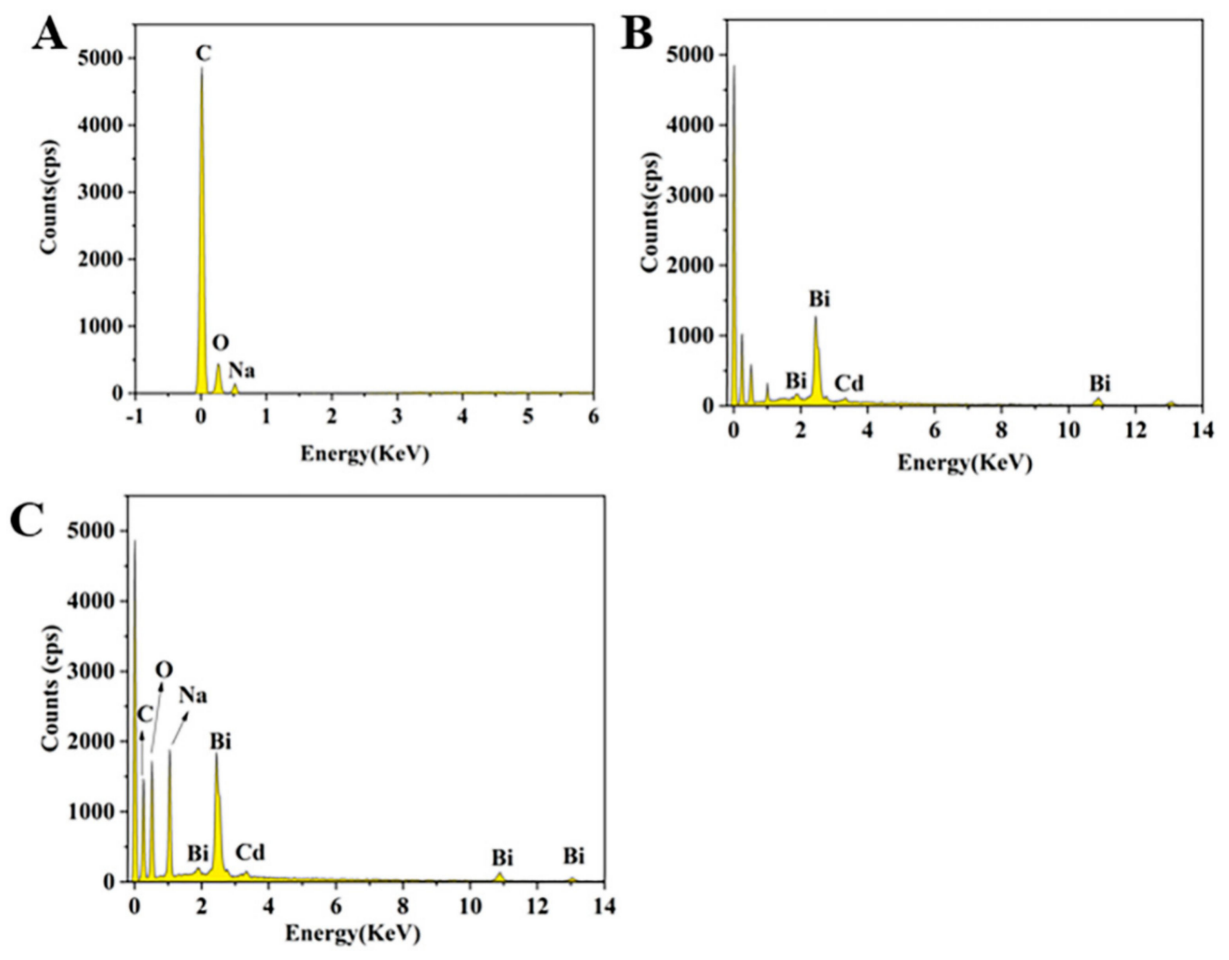
EDS spectra of CMC-GCE (**A**), bare GCE after electrodeposition of cadmium and bismuth (**B**), and CMC-GCE after electrodeposition of cadmium and bismuth (**C**).

To study the synergetic effects of both CMC and bismuth on cadmium enrichment, firstly a series of scan electron microscopy (SEM) photographs were obtained as shown in Figure 2. In Figure 2A, the bare GCE surface can be seen to be smooth, without any modification of CMC or deposition of cadmium and bismuth. In Figure 2B, it can be seen that the morphology turns out to be strip-shaped, indicating the successful modification of the bare GCE with CMC. In Figure 2C, there appear many irregular and small particles on the bare GCE surface. This phenomenon can be attributed to the co-deposition of both cadmium ions and bismuth ions. While in Figure 2D, a larger number of smaller particles with regular shapes can be observed on CMC-GCE surface, reflecting that cadmium ions can be better electrodeposited together with bismuth ions onto the electrode surface with the presence of CMC. At the same time, elemental compositions were examined by energy dispersive spectroscopy (EDS). As shown in Figure 3A, C, O and Na elementals for CMC-GCE can be clearly found. In Figure 3B or C, both cadmium and bismuth ions can be detected on the bare GCE or CMC-GCE surface, after electrodeposition has happened in NaAc/HAc buffer solution containing 0.2 μmol/L of Cd^2+^ and Bi^3+^; while on the CMC-GCE surface, more cadmium and bismuth ions can be observed. These results are consistent with those of SEM analyses. Therefore, both SEM and EDS studies indicate that CMC has a good film-forming talent that is favorable for the smooth modification of GCE; moreover, it has a good adsorption/enrichment ability for better electrodeposition of both cadmium and bismuth ions. This may be because CMC contains a large amount of hydroxyl and carboxyl groups, which help to attract more cadmium ions as well as bismuth ions onto the electrode surface through synergetic coordination as described in Scheme 1.

### 3.3. Construction and Investigation of the Proposed Cadmium Sensor

**Figure 4:**
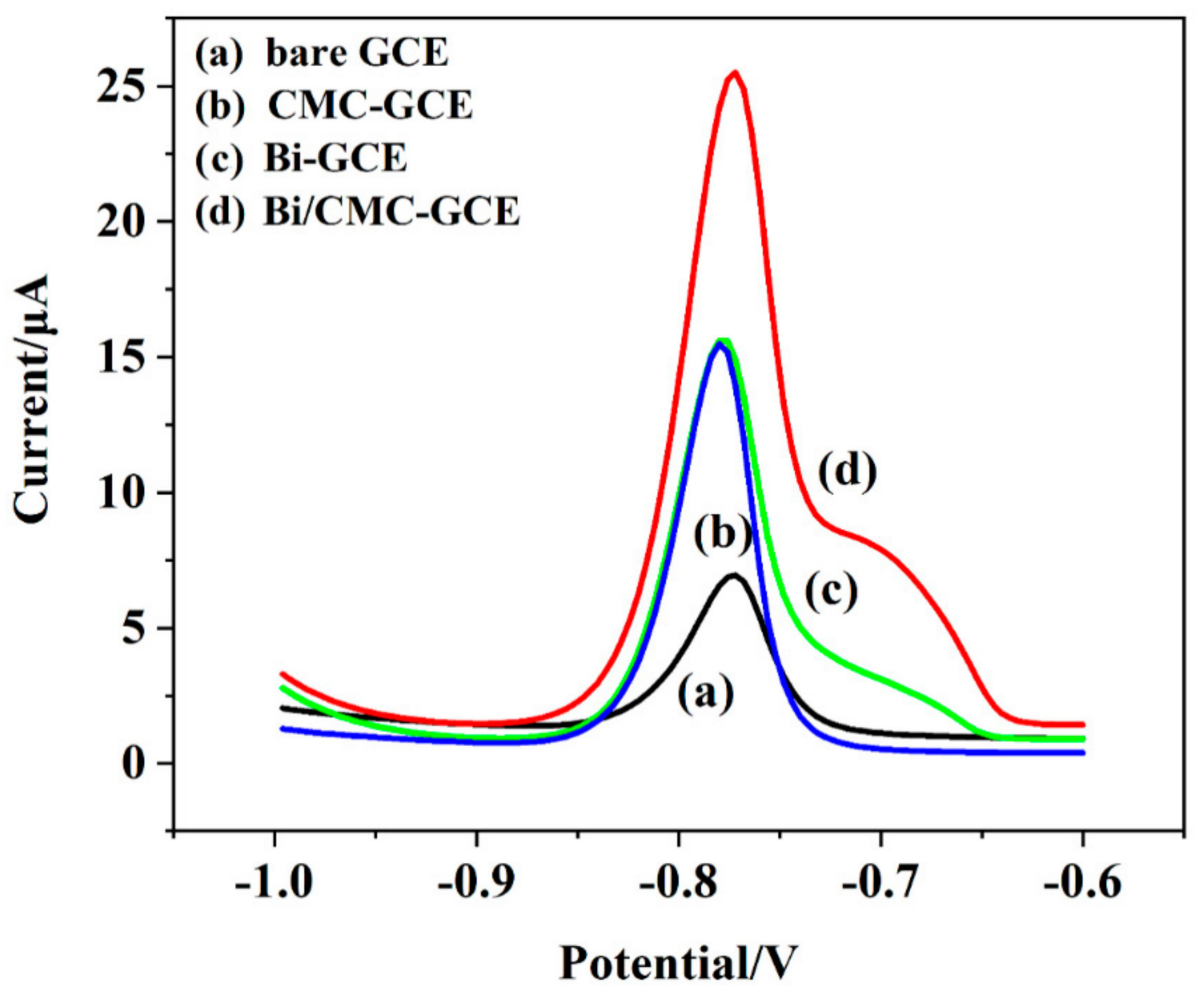
Differential pulse anodic stripping voltammograms for 0.5 μmol/L of Cd^2+^ in NaAc/HAc (0.10 mol/L, pH 4.5) buffer solution at the surface of bare GCE (**a**), CMC-GCE (**b**), Bi-GCE (**c**) and Bi/CMC-GCE (**d**). Deposition potential: −1.2 V; accumulation time 150 s; scan rate: 50 mV·s^−1^.

The proposed cadmium (II) (Cd^2+^) sensor was constructed according to the strategy as shown in Scheme 1 and was investigated by DPASV. The results are shown in Figure 4, including DPASV voltammograms of Cd^2+^ obtained at different electrodes: bare GCE (a), CMC-GCE (b), Bi-GCE (c), and Bi/CMC-GCE (d), respectively. As can be seen from Figure 4, (i) stripping signals and good peak shapes can be clearly observed for all of electrodes (curve a–d), showing that herein DPASV is the very technique that is suitable for the performance investigations on this proposed Cd^2+^ sensor, and a quite simple drop-casting method or the fairly convenient in situ electro-deposition method can respectively modify CMC or bismuth ions (coexisting with target cadmium ion in the sample solution) onto the GCE surface; (ii) both of the stripping signals obtained at CMC-GCE and Bi-GCE (curve b and c) are larger than that of the bare GCE, indicating that the modification of the bare GCE with either sodium carboxymethyl cellulose or bismuth film can enhance the signal response to Cd^2+^; (iii) it is quite obvious that the largest stripping signal appears at Bi/CMC-GCE (curve d), demonstrating that the modification of the bare GCE with both sodium carboxymethyl cellulose and bismuth film can best improve the electrochemical performance of the sensor, which finally amplifies the signal response to a large extent and provides extraordinary possibilities for the detection of trace cadmium ions. This phenomenon may be attributed to the synergetic effect of CMC and bismuth ions. Based on good film-forming and metal-chelating properties, CMC can efficiently drive bismuth ions and also cadmium ions (their alloy) to in situ deposit together upon the electrode surface, leading to an extra and considerable increase (curve d) in the already well-defined stripping signal (curve b or c). All of the results reflect the successful fabrication of the new cadmium (II) sensor according to the proposed strategy as described in Scheme 1. Therefore, this new sensor, based on a CMC-modified GCE by a simple drop-casting method followed by in situ bismuth deposition (Bi/CMC-GCE), has great potential for the detection of trace cadmium residue in an easy way with high sensitivity.

### 3.4. Optimization of Experimental Conditions

To further improve the performance of the prepared sensor for the detection of Cd^2+^ at a very low level, several parameters that can affect the DPASV signals were sequentially investigated, including the pH value of the buffer solution, the accumulation time, the deposition potential and the concentration of CMC or Bi^3+^ solution. The experimental parameters were optimized as follows.

#### 3.4.1. The pH Value of the Buffer Solution

**Figure 5:**
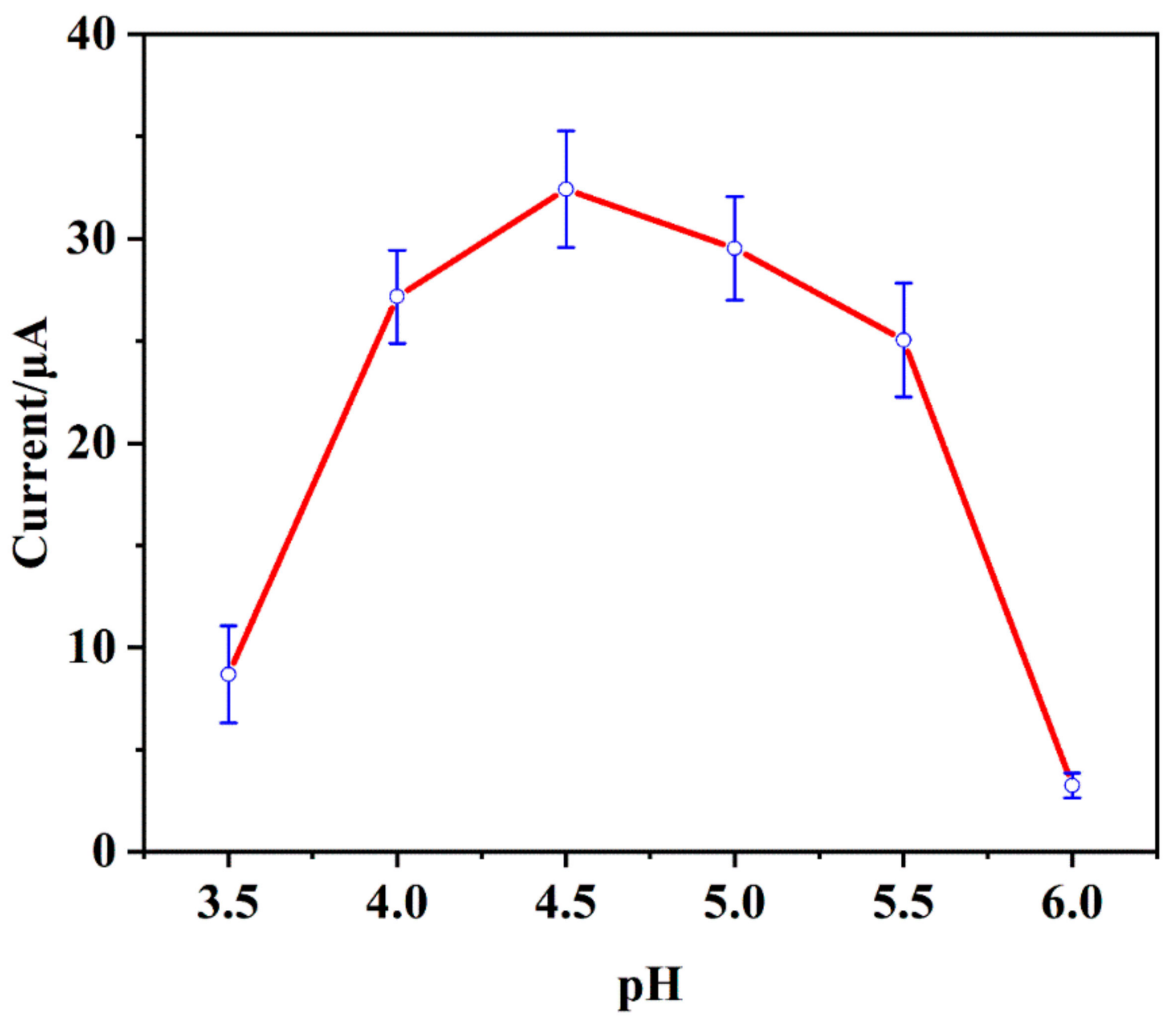
Effect of pH on the stripping peak current of 0.5 μmol/L of Cd^2+^ at Bi/CMC-GCE in 0.10 mol/L of NaAc/HAc buffer solution. The investigated pH values were 3.5, 4.0, 4.5, 5.0, 5.5, and 6.0. Deposition potential: −1.2 V; accumulation time: 150 s; scan rate: 50 mV·s^−1^.

Firstly, the influence of the pH value of the buffer solution (NaAc/HAc, 0.1 mol/L) on the stripping peak current response to 0.5 μmol/L of Cd^2+^ was studied in a pH range from 3.5 to 6.0 by DPASV and results are shown in Figure 5. It can be seen that the stripping peak current of Cd^2+^ firstly gradually rose as the pH increased from 3.5 to 4.5, but then gradually dropped as the pH increased from 4.5 to 6.0. Obviously, when pH was 4.5, the current signal reached its maximum. This phenomenon could be explained by the fact that on the one hand, there is a competition between metal ions and protons for active sites on the electrode surface since they are both positive-charged and will naturally repel each other; and on the other hand, metal ions are prone to hydrolyze at a relatively high pH [39,40]. Therefore, when pH < 4.5, the higher the proton concentration in the buffer was (the lower the pH value was), the bigger the electrostatic repulsion between protons and metal ions would be, and the less metal ions could be adsorbed and deposited onto the electrode surface so that a lower stripping signal could be obtained. When pH > 4.5, the higher the pH value was, the more metal ions would undergo hydrolysis, and also a lower stripping signal could be observed. Thus, as can be easily seen from Figure 5, the optimal pH value for this new Cd^2+^ sensor is 4.5, at which all the following electrochemical measurements will be carried out.

#### 3.4.2. Accumulation Time

**Figure 6:**
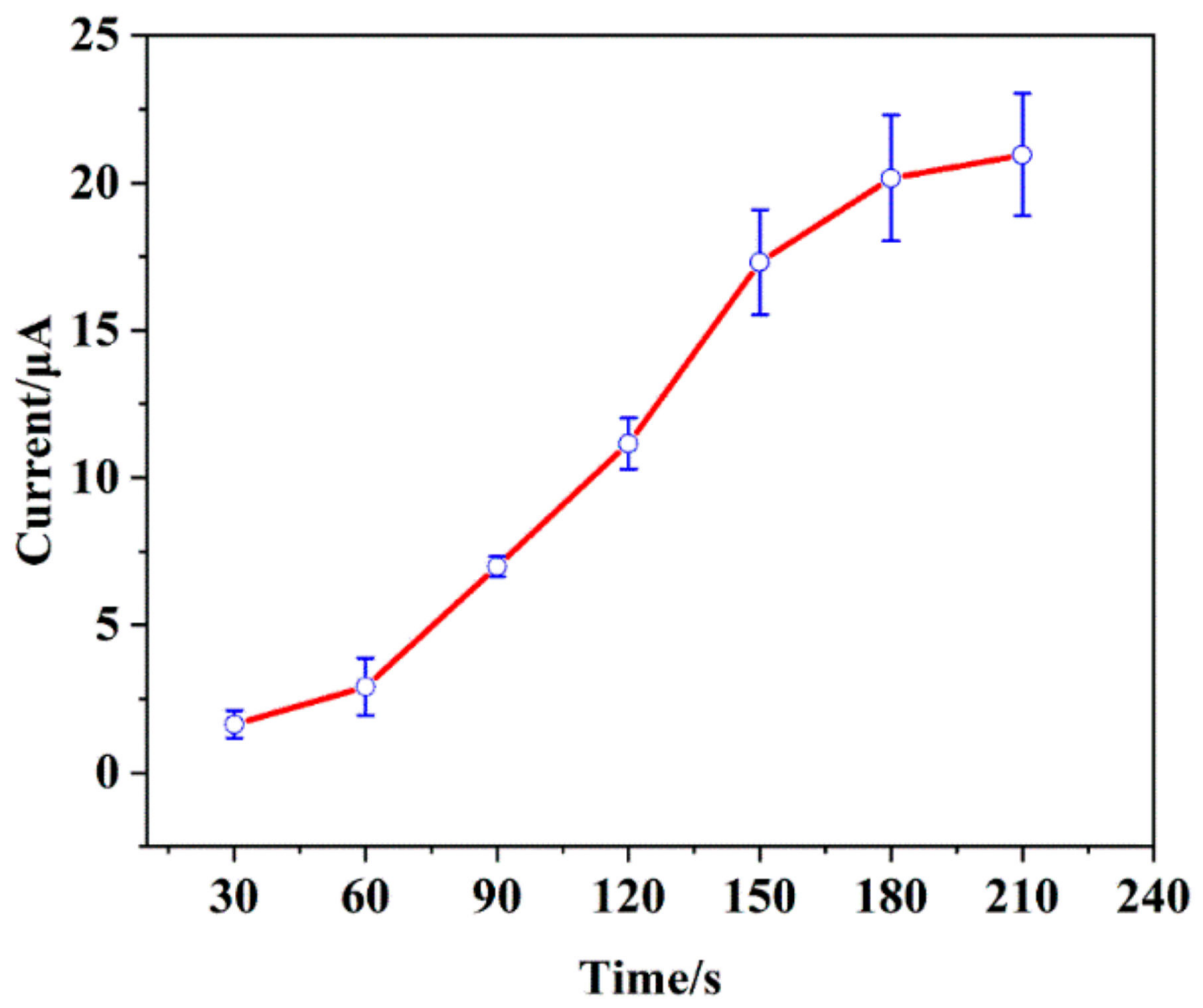
Effect of accumulation time on the stripping peak current of 0.5 μmol/L of Cd^2+^ at Bi/CMC-GCE in NaAc/HAc buffer solution (0.10 mol/L, pH 4.5). The investigated accumulation times were 30 s, 60 s, 90 s, 120 s, 150 s, 180 s, and 210 s. Deposition potential, −1.2 V; scan rate: 50 mV·s^−1^.

Secondly, under the optimal pH condition (pH 4.5) adjusted by 0.10 mol/L of NaAc/HAc solution, the effect of accumulation time on the stripping signal response to 0.5 μmol/L of Cd^2+^ was investigated in a time range from 30 s to 210 s, and results are shown in Figure 6. The stripping peak current kept rising with the pro-long of accumulation time; quickly at first from 30 s to 180 s, but then slowly from 180 s to 210 s. This phenomenon may be attributed to the fact that there are lots of functional groups (hydroxyl and carboxyl methyl groups) in the polymer chains of CMC to endow the electrode surface with lots of active sites, which greatly facilitates the deposition of metal ions from the solution onto the electrode surface as much as possible. Before the surface reached saturation herein from 30 s to 180 s, given more time, more metal ions could be immobilized upon the electrode, so that the stripping signal went up at a higher rate; whilst after the surface reached saturation, along with the increase of time herein from 180 s to 210 s, the amount of the deposited metal ions could not be raised as before (appeared to be stabilized), leading to a slower growth in the stripping signal. By a comprehensive consideration of both effect and efficiency, therefore, 180 s is chosen as a best suitable accumulation time for all the following experiments.

#### 3.4.3. Deposition Potential

**Figure 7:**
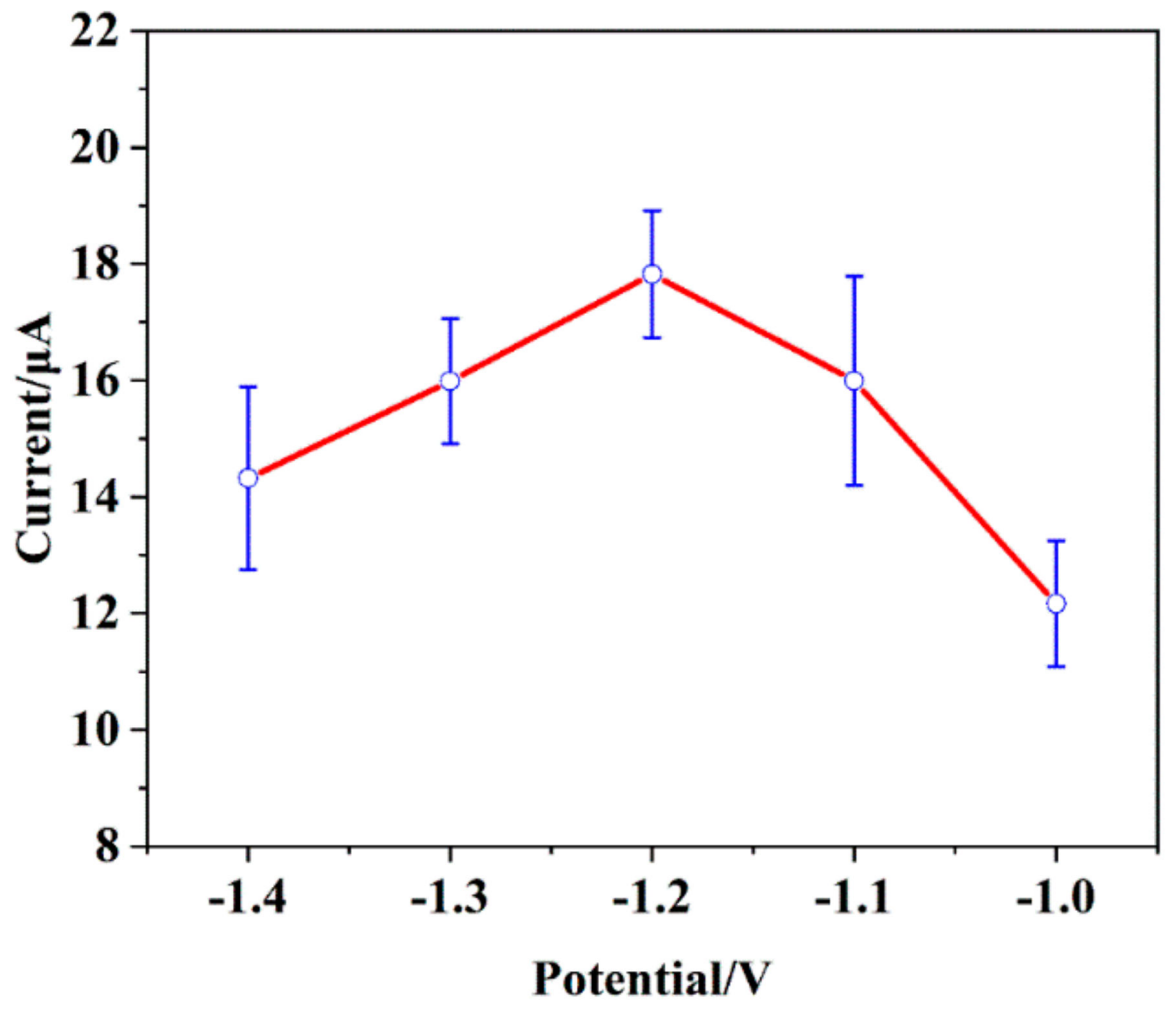
Effect of deposition potential on the stripping peak current of 0.5 μmol/L of Cd^2+^ at Bi/CMC-GCE in NaAc/HAc buffer solution (0.10 mol/L, pH 4.5). The investigated deposition potentials were −1.4 V, −1.3 V, −1.2 V, −1.1 V, and −1.0 V. Accumulation time 180 s; scan rate: 50 mV·s^−1^.

Thirdly, at pH 4.5 with an accumulation time of 180 s, the influence of the deposition potential on the stripping signal response to 0.5 μmol/L of Cd^2+^ was studied in a range from −1.0 V to −1.4 V, and results are shown in Figure 7. It can be seen that the stripping peak current for target Cd^2+^ gradually increased when the negative deposition potential shifted from −1.0 V to −1.2 V, and then gradually decreased from −1.2 V to −1.4 V. Apparently, the current reached its maximum under a deposition potential of −1.2 V. This can be explained by the higher negative potential hydrogen evolution and the incomplete reduction of the bismuth-metal alloy at a more positive potential [2,41]. Therefore, the optimal deposition potential for this new Cd^2+^ sensor can be determined to be −1.2 V.

#### 3.4.4. The Concentration of Sodium Carboxymethyl Cellulose (CMC)

**Figure 8:**
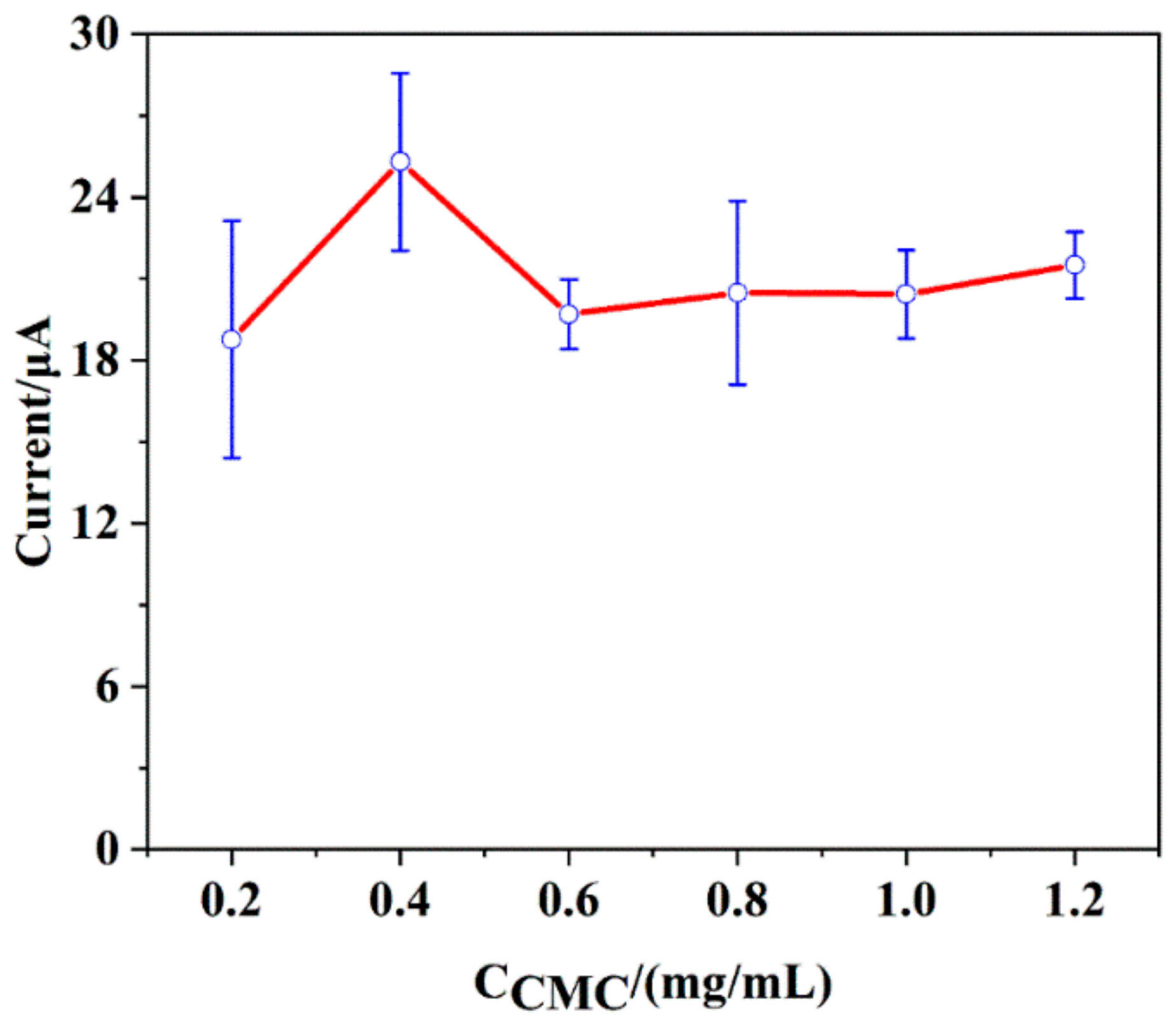
Effect of concentration of CMC on the stripping peak current of 0.5 μmol/L of Cd^2+^ at Bi/CMC-GCE in NaAc/HAc buffer solution (0.10 mol/L, pH 4.5). The investigated concentration of CMC were 0.2, 0.4, 0.6, 0.8, 1.0, and 1.2 mg/mL. Accumulation time 180 s; deposition potential: −1.2 V; scan rate: 50 mV·s^−1^.

Fourthly, at pH 4.5 under a deposition potential of –1.2 V along with an accumulation time of 180 s, the effect of CMC concentration on the stripping signal response to 0.5 μmol/L of Cd^2+^ was investigated in a CMC concentration range from 0.2 to 1.2 mg/mL, and results are shown in Figure 8. The stripping peak current firstly rose along with the increase of CMC concentration from 0.2 to 0.4 mg/mL, but then dropped rapidly when the concentration went on increasing from 0.4 to 0.6 mg/mL, and finally almost remained unchanged when further increasing CMC concentration from 0.6 to 1.2 mg/mL. This phenomenon might be explained as follows: when the CMC concentration firstly rose from 0.2 to 0.4 mg/mL, the amounts of the hydroxyl and carboxyl methyl groups on the electrode surface would increase, so that more metal ions could be adsorbed and deposited upon the modified electrode, leading to an enhancement of the current signal; meanwhile, the improvement of CMC concentration also added the thickness of the CMC film that was modified onto the electrode surface, which reduced the electrode conductivity and so caused a decrease in the current signal, as observed from 0.4 to 0.6 mg/mL; then, from 0.6 to 1.2 mg/mL, when the CMC concentration continued to increase, the electrode surface was finally saturated with active hydroxyl/carboxyl groups as well as deposited metal ions, so that the current signal eventually remained almost stable [42]. Therefore, it can be concluded that the concentration of sodium carboxymethyl cellulose most suitable for the modification of bare GCE is 0.4 mg/mL.

#### 3.4.5. The Concentration of Bismuth Ion Solution (Bi^3+^)

**Figure 9:**
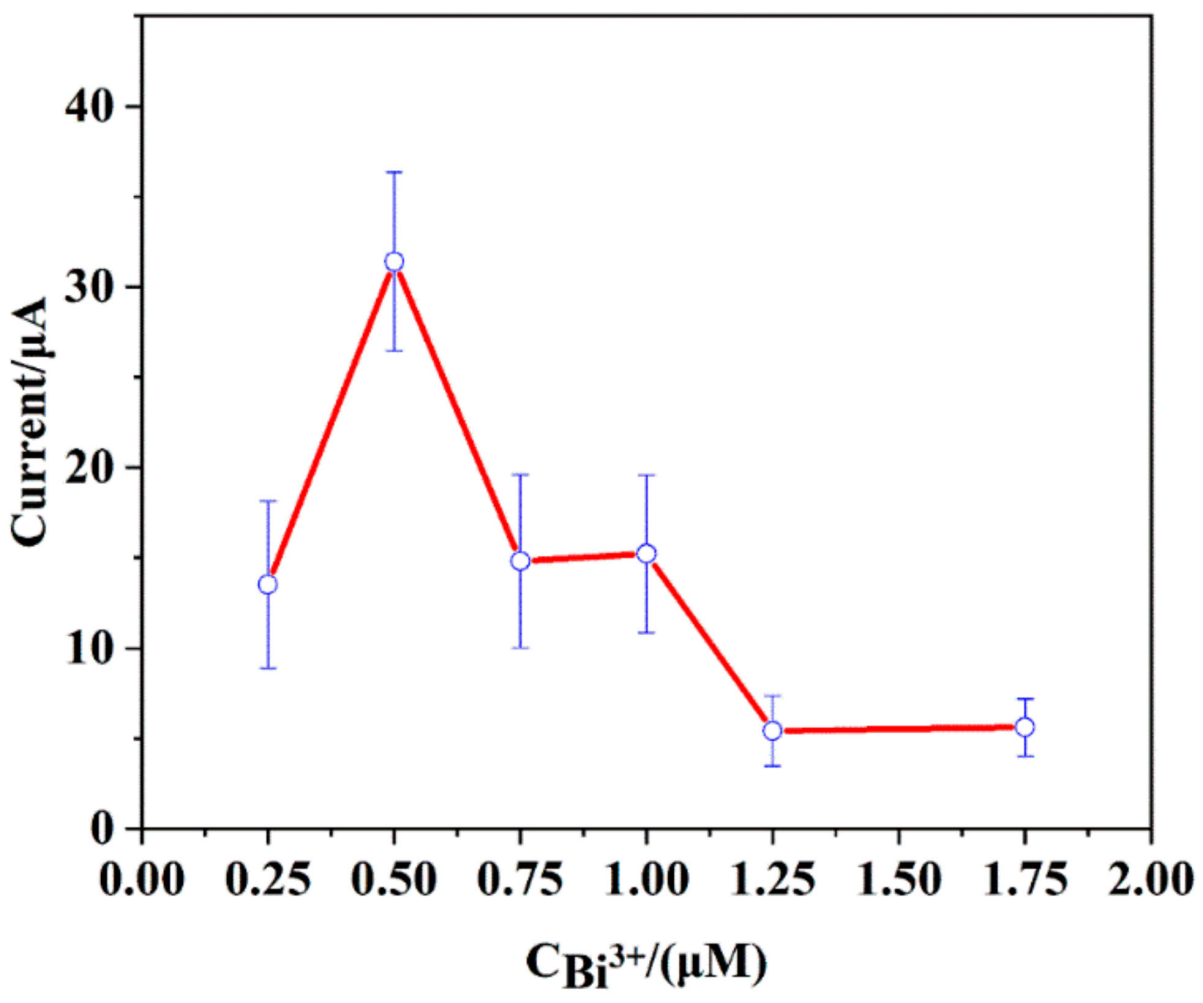
Effect of concentration of Bi^3+^ solution on the stripping peak current of 0.5 μmol/L of Cd^2+^ at Bi/CMC-GCE in NaAc/HAc buffer solution (0.10 M, pH 4.5). The investigated concentration of Bi^3+^ solution were 0.25, 0.50, 0.75, 1.00, 1.25, 1.50, 1.75 μmol/L. CMC solution: 0.4 mg/mL; accumulation time 180 s; deposition potential: −1.2 V; scan rate: 50 mV·s^−1^.

Finally, using CMC solution of 0.4 mg/mL to modify the bare GCE and then tested at pH 4.5 under a deposition potential of −1.2 V along with an accumulation time of 180 s, the effect of Bi^3+^ concentration on the stripping signal response to 0.5 μmol/L of target Cd^2+^ was investigated in a Bi^3+^ concentration range from 0.25 to 1.75 μmol/L, and results are shown in Figure 9. The stripping peak currents firstly increased when the Bi^3+^ concentrations increased from 0.25 to 0.5 μmol/L, but then as a whole decreased when the concentration further increased from 0.5 to1.75 μmol/L. It might be attributed to the dual characters of bismuth ion. On the one hand, bismuth ion can form an alloy with cadmium ion to help the latter to be synchronously electrodeposited upon the electrode surface, so increasing Bi^3+^ concentration to a certain extent will surely increase the stripping signal of target Cd^2+^, as observed from 0.25 to 0.5 μmol/L. On the other hand, bismuth ions will compete with cadmium ions for the active site on the electrode surface [43], so the further increase of Bi^3+^ concentration will in turn reduce the amount of cadmium ions that can be deposited upon the electrode surface, resulting in a decrease in the stripping signal of target Cd^2+^, as observed from 0.5 to 1.75 μmol/L. Therefore, the optimal concentration of bismuth ion solution, which will be in situ electrodeposited together with target Cd^2+^ upon the surface of the CMC-modified electrode, can be determined to be 0.5 μmol/L. 

### 3.5. Sensitivity of the Proposed Cadmium Sensor

**Figure 10:**
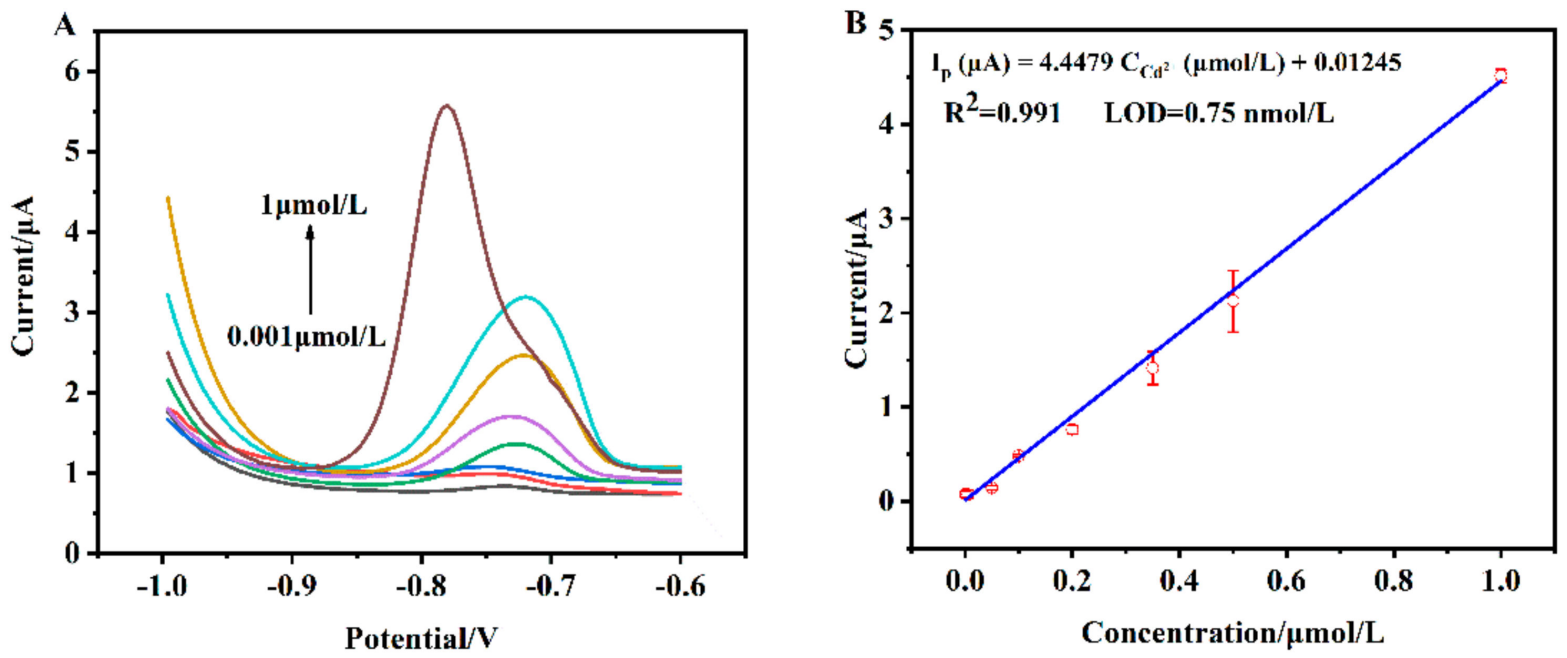
(**A**) Differential pulse anodic stripping voltammograms for different concentrations of Cd^2+^ solution at Bi/CMC-GCE in NaAc/HAc buffer solution (0.10 mol/L, pH 4.5) containing 0.5 μmol/L of Bi^3+^. The concentrations of Cd^2+^ solution were 0.001, 0.005, 0.05, 0.1, 0.2, 0.35, 0.5 and 1 μmol/L, respectively, from bottom to top. (**B**) The corresponding calibration curve of stripping peak current versus concentration. CMC solution: 0.4 mg/mL; accumulation time 180 s; deposition potential: −1.2 V; scan rate: 50 mV·s^−1^.

Under the optimized experimental conditions (CMC solution: 0.4 mg/mL; bismuth ion solution: 0.5 μmol/L; pH 4.5; deposition potential: −1.2 V; accumulation time: 180 s), the stripping peak current values of the proposed sensor based on Bi/CMC-GCE in solutions with different concentration of target Cd^2+^ were investigated via DPASV (Figure 10). As shown in Figure 10A, the stripping peak current rose along with the increase of Cd^2+^ concentration from 0.001 μmol/L to 1 μmol/L. The corresponding calibration curve was plotted as shown in Figure 10B. It can be obviously seen that there is a good linear relationship between values of the stripping peak current and target Cd^2+^ concentrations across a range of 0.001 μmol/L to 1 μmol/L, with correlation equation I_p_ (μA) = 4.4479 Cd^2+^ (μmol/L) + 0.01245 and correlation coefficient of 0.991. The limit of detection (LOD) was calculated to be 0.75 nmol/L at a signal-to-noise ratio of 3 (S/N=3). These results demonstrate that the proposed Cd^2+^ sensor based on Bi/CMC-GCE can have a high sensitivity for the following merits: distinct film-forming talent of CMC, well-defined stripping signal, and wide negative potential window of bismuth ions, as well as both of their chelating capabilities with cadmium ions, and especially their synergetic effects which amplify the stripping signal to be big enough for sensitive determination of trace cadmium. As can be seen from Table 1, compared with some Cd^2+^ electrochemical sensors reported in recent years which used various kinds of composites mainly containing bismuth ion or carbon materials to modify electrodes, our work presents an excellent sensing platform that can be fabricated by low-cost and eco-friendly materials in a quite simple way to show good sensitivity for trace Cd^2+^ detection with a low LOD and wide dynamic range.

### 3.6. Interference Study

**Figure 11:**
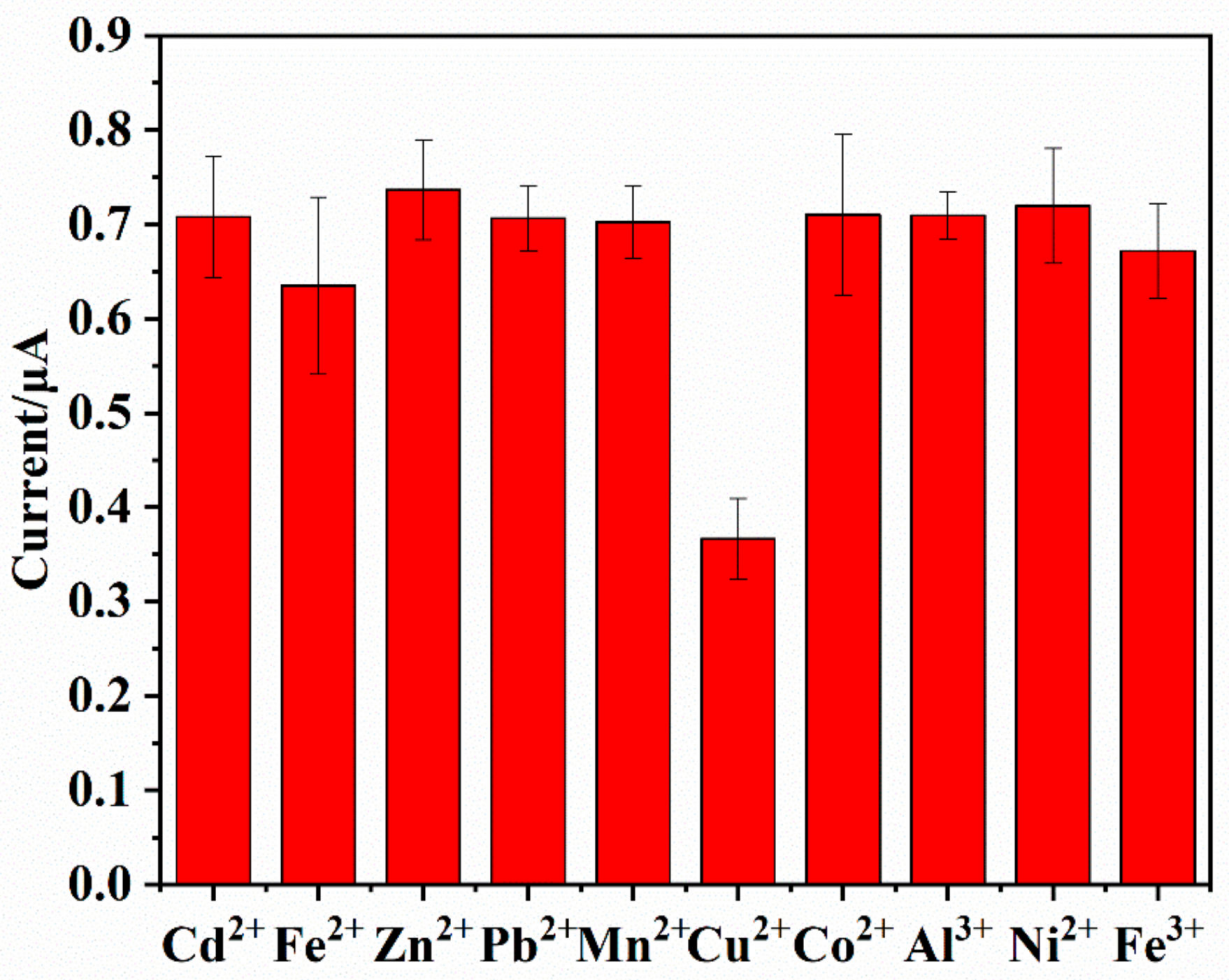
Interferences caused by co-existing cations (5 μmol/L) on the stripping peak current of 0.2 μmol/L Cd^2+^. All measurements were carried out under the optimized experimental conditions. CMC solution: 0.4 mg/mL; bismuth ion solution: 0.5 μmol/L; pH 4.5; deposition potential: −1.2 V; accumulation time: 180 s; scan rate: 50 mV·s^−1^.

The influence of the co-existing ions on the determination of Cd^2+^ was investigated under the optimum conditions by adding various ions (25-fold) into standard solution containing 0.2 μmol/L of Cd^2+^. As shown in Figure 11, 25-fold Fe^2+^, Zn^2+^, Pb^2+^, Mn^2+^, Co^2+^, Al^3+^, Ni^2+^, Fe^3+^ brought about small changes on the signal of Cd^2+^ under the ±5.0% tolerated ratios, implying that these normally encountered metal ions cause no significant interference in Cd^2+^ detection. However, in the presence of 25-fold Cu^2+^, the peak current of cadmium ion declined sharply. This phenomenon didn’t only appear in our present work. It was reported by other researchers and could be attributed to the competition between Cu^2+^ and Bi^3+^ [53,54]. They would fight not only for target Cd^2+^ to form intermetallic compounds (metal alloy), but also for active sites on the electrode surface. However, this interference caused by Cu^2+^ could be easily eliminated by adding ferrocyanide in the tested solution to form a stable and insoluble copper-ferrocyanide complex. Therefore, our proposed sensor exhibits good anti-interference performance and is suitable for the detection of target Cd^2+^ with high selectivity.

### 3.7. Reproducibility and Repeatability of the Proposed Sensor

**Figure 12:**
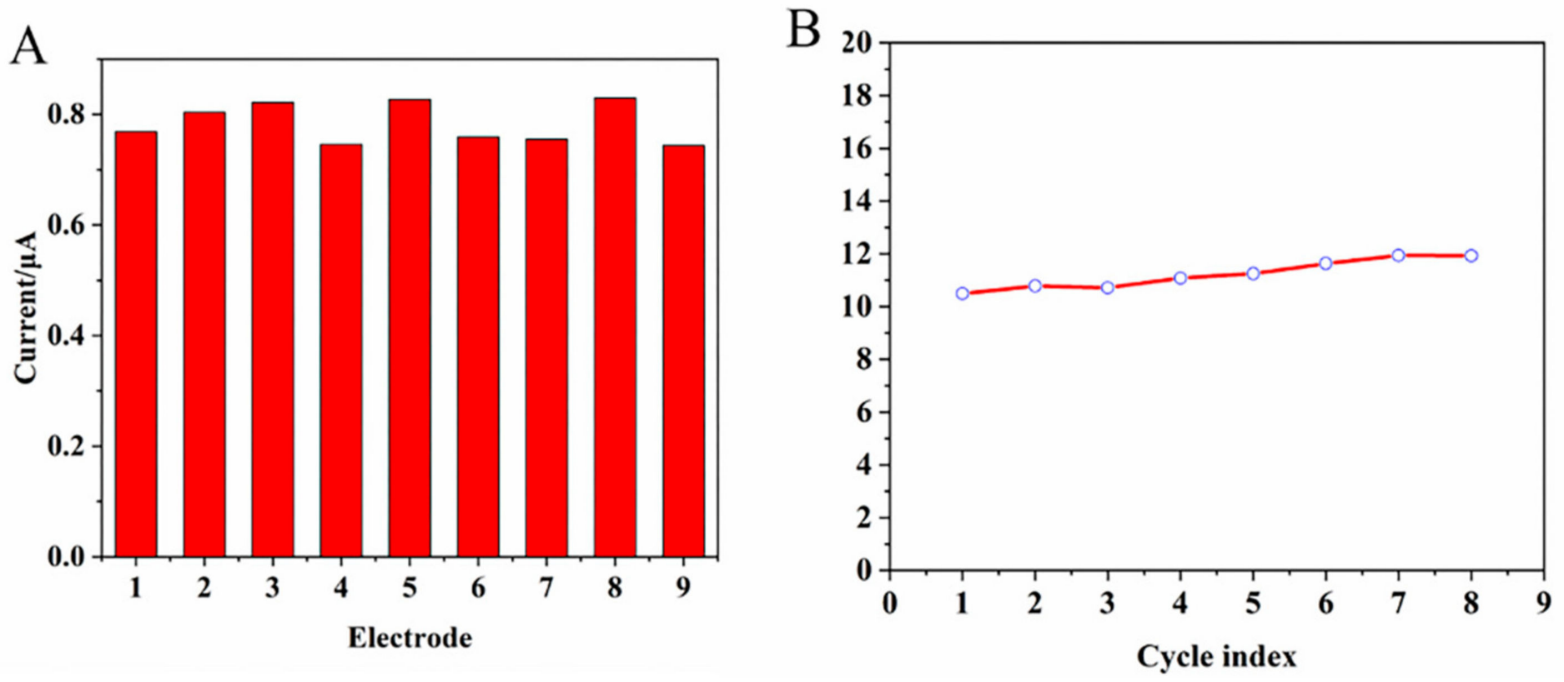
(**A**) Stripping peak currents obtained on nine electrodes (Bi/CMC-GCE) for 0.5 μmol/L of Cd^2+^ solution. (**B**) The repeatability of the proposed sensor by repeatedly measuring the stripping peak current eight times on the same modified electrode (Bi/CMC-GCE) in 0.2 μmol/L of Cd^2+^ solution. All measurements were carried out under the optimized experimental conditions. CMC solution: 0.4 mg/mL; bismuth ion solution: 0.5 μmol/L; pH 4.5; deposition potential: −1.2 V; accumulation time: 180 s; scan rate: 50 mV·s^−1^.

In order to further study the performances of the developed Cd^2+^ sensor, both reproducibility and repeatability were tested. Firstly, reproducibility was evaluated by measuring the stripping peak currents under the same optimal experimental conditions on 9 freshly modified electrodes in 0.5 μmol/L Cd^2+^ solution. At the same time, repeatability was validated by repeatedly measuring the stripping peak current eight times on the same modified electrode in 0.2 μmol/L Cd^2+^ solution. Results are shown in Figure 12. From Figure 12A for nine independent electrodes, an acceptable value of the relative standard deviation (RSD) was 4.97% for 0.5 μmol/L Cd^2+^ solution, demonstrating the good reproducibility of the proposed sensor. From Figure 12B for the same electrode, the RSD value was 4.63% for 0.2 μmol/L Cd^2+^ solution in eight repeated measurements, confirming the good repeatability of the proposed sensor. 

### 3.8. Analysis of Tap Water Samples

Under the optimal analytical conditions, tap water was used to prepare actual samples that contained Cd^2+^ salt by using standard addition method, to further investigate the application performance of the proposed sensor. Results are shown in Table 2. Firstly, no detectable Cd^2+^ was found in tap water. Then, with the addition of known amounts of Cd^2+^, the developed Bi/CMC-GCE exhibited good recovery results of 93.0%–110%. Thus, the present sensing platform based on Bi/CMC-GCE shows great prospects on the accurate detection of Cd^2+^ in real water samples.

## 4. Conclusions

In this study, an electrochemical sensor, based on the in situ electrodeposition of bismuth ions onto the surface of a glassy carbon electrode modified by CMC in a simple drop-casting way, was successfully constructed to detect cadmium ions at a sub-nanomolar level by differential pulse anodic stripping voltammetry. Results show that this new electrochemical platform exhibits a good linear stripping signal response to target cadmium ions in a concentration range of 0.001 μmol/L–1 μmol/L, with a limit of detection of 0.75 nmol/L, under the optimized conditions, with a concentration of CMC solution of 0.4 mg/mL and a concentration of bismuth ion solution of 0.5 μmol/L at pH 4.5 under a deposition potential of −1.2 V along with an accumulation time of 180 s. This low-cost and eco-friendly cadmium ion sensor is easy to prepare, and exhibits good reproducibility and repeatability as well as excellent anti-interference for normally co-existed metal ions. Meanwhile, this new cadmium electrochemical sensor based on the synergetic sensing effect of bismuth and CMC shows a promising application prospect in natural samples with a good recovery from 93.0% to 110% in tap water which is polluted by Cd^2+^ salt.

## Figures and Tables

**Table 1:** Comparison of some cadmium electrochemical sensors reported previously.

Modified Electrodes	Methods	Linear Range (μmol/L)	LOD (μmol/L)	Ref.
GCE+MMT+TBAB (partial)/M	SWASV a	0.2–2.5	0.072	[44]
Bi-MGF/GCE	DPASV b	0.017–0.625	0.004	[45]
MC/MA/Nafion/GC	SWASV	0.05–0.3	0.024	[46]
PPE-GCE	SWASV	0.017–0.89	0.007	[47]
CNT-SO3H/RhB-LB/GCE	SWASV	0.1–1.2	0.08	[48]
Bi/GaN	SWASV	0.009–1.334	0.0027	[49]
SAM electrode	DPV c	15–65	4.5	[50]
Bi-N-HPCSs/GCE	DPASV	0.004–1.33	0.001	[51]
N,S-PC-Nafion/Bi/GCE	DPASV	0.035–0.714	0.0008	[52]
Bi/CMC-GCE	DPASV	0.001–1	0.00075	This work

^a^ SWASV: square wave anodic stripping voltammetry; ^b^ DPASV: differential pulse anodic stripping voltammetry; ^c^ DPV: differential pulse voltammetry

**Table 2:** Results for the determination of Cd^2+^ in tap water samples.

Sample	Added (μmol/L)	Found (μmol/L)	Recovery (%)
Tap water	-	-	-
	0.01	0.011	110
	0.02	0.021	105
	0.10	0.09	93.0
